# Docosahexaenoic Acid Conjugation Enhances Distribution and Safety of siRNA upon Local Administration in Mouse Brain

**DOI:** 10.1038/mtna.2016.50

**Published:** 2016-08-09

**Authors:** Mehran Nikan, Maire F Osborn, Andrew H Coles, Bruno MDC Godinho, Lauren M Hall, Reka A Haraszti, Matthew R Hassler, Dimas Echeverria, Neil Aronin, Anastasia Khvorova

**Affiliations:** 1 RNA Therapeutics Institute, University of Massachusetts Medical School, Worcester, Massachusetts, USA; 2 Department of Molecular Medicine, University of Massachusetts Medical School, Worcester, Massachusetts, USA; 3 Department of Medicine, University of Massachusetts Medical School, Worcester, Massachusetts, USA

**Keywords:** drug delivery, neurodegenerative disease, siRNA

## Abstract

The use of siRNA-based therapies for the treatment of neurodegenerative disease requires efficient, nontoxic distribution to the affected brain parenchyma, notably the striatum and cortex. Here, we describe the synthesis and activity of a fully chemically modified siRNA that is directly conjugated to docosahexaenoic acid (DHA), the most abundant polyunsaturated fatty acid in the mammalian brain. DHA conjugation enables enhanced siRNA retention throughout both the ipsilateral striatum and cortex following a single, intrastriatal injection (ranging from 6–60 μg). Within these tissues, DHA conjugation promotes internalization by both neurons and astrocytes. We demonstrate efficient and specific silencing of Huntingtin mRNA expression in both the ipsilateral striatum (up to 73%) and cortex (up to 51%) after 1 week. Moreover, following a bilateral intrastriatal injection (60 μg), we achieve up to 80% silencing of a secondary target, Cyclophilin B, at both the mRNA and protein level. Importantly, DHA-hsiRNAs do not induce neural cell death or measurable innate immune activation following administration of concentrations over 20 times above the efficacious dose. Thus, DHA conjugation is a novel strategy for improving siRNA activity in mouse brain, with potential to act as a new therapeutic platform for the treatment of neurodegenerative disorders.

## Introduction

The clinical use of siRNA therapeutics for the treatment of neurodegenerative disease is limited by a lack of reliable and nontoxic delivery routes to the central nervous system (CNS). siRNAs do not cross the blood–brain barrier and must therefore be directly injected or infused into the cerebrospinal fluid (CSF) or brain.^1,2,3,4^ However, many neurodegenerative disorders affect multiple regions of the brain, particularly the striatum and cortex, requiring broad distribution from the site of administration.^5^ Unmodified siRNAs directly injected into the brain are rapidly degraded or cleared from the CSF^6^ and continuous, large dose infusions are required to achieve local gene silencing.^4,7^ Typical lipid- and polymer-based siRNA formulations that improve cellular uptake show pronounced toxicity and inflammatory response when administered in the brain.^8,9,10^

We recently reported that cholesterol-conjugated hydrophobically modified siRNAs (Chol-hsiRNAs) efficiently silence Huntingtin (*Htt*) mRNA when injected directly into the striatum.^11^ However, these compounds exhibit a steep gradient of diffusion from the injection site. This limits the utility of this platform in larger brains, and thus, its potential as a therapeutic modality. We explored if conjugation to less hydrophobic, brain-enriched lipids could improve siRNA distribution and minimize immunogenicity *in vivo*. Here, for the first time, we report the synthesis, and *in vitro* and *in vivo* efficacy, of a docosahexaenoic acid-hsiRNA conjugate (DHA-hsiRNA) targeting *Htt* mRNA.

DHA is an omega-3 fatty acid and the principal fatty acid in the mammalian brain. DHA and its metabolites are involved in a variety of essential biological processes, including neurogenesis and neurotransmission.^12^ In the brain, DHA is primarily found esterified into the phospholipid membranes of neural cells.^13,14^ DHA-containing lipids are substrates for hydrolyzing and oxidative enzymes (*e.g.*, cyclooxygenase and lipooxygenase), which are capable of generating a range of potent neuroprotective metabolites, including neuroprotectin and resolvin.^15,16,17^ Orally administered omega-3 fatty acids have shown anti-inflammatory and neuroprotective properties in the clinic.^18^ This is of particular interest from an RNAi perspective, where immune response and toxicity are major concerns.^19^ Polyunsaturated fatty acids have also been investigated for potential antitumor effects, either alone or conjugated to chemotherapeutic drugs.^20,21^

There are two previous reports of polyunsaturated fatty acid conjugation to therapeutic RNAs. Willibald *et al.* conjugated an anandamide to a partially modified siRNA, demonstrating mRNA silencing in human immune B (BJAB) and rat basophilic leukemia (RBL-2H3) cells *in vitro*.^22^ Felber *et al.* synthesized FANA-modified antisense oligonucleotides conjugated to docosanoic acid, DHA, and cholesterol. These conjugates showed marginal activity in human prostrate cancer (PC-3) cells and were completely inhibited in the presence of 20% serum.^23^ These compounds were not tested in primary cells or animals, supporting the notion that compound behavior in transformed cell lines is not necessarily predictive of compound distribution and activity *in vivo*.

The main objective of this study was to perform a comprehensive and systematic analysis of the efficacy, distribution, and toxicity of a DHA-hsiRNA conjugate in primary cortical neurons as well as in mouse brain.

## Results

### Design and synthesis of DHA-hsiRNA

**Figure 1:**
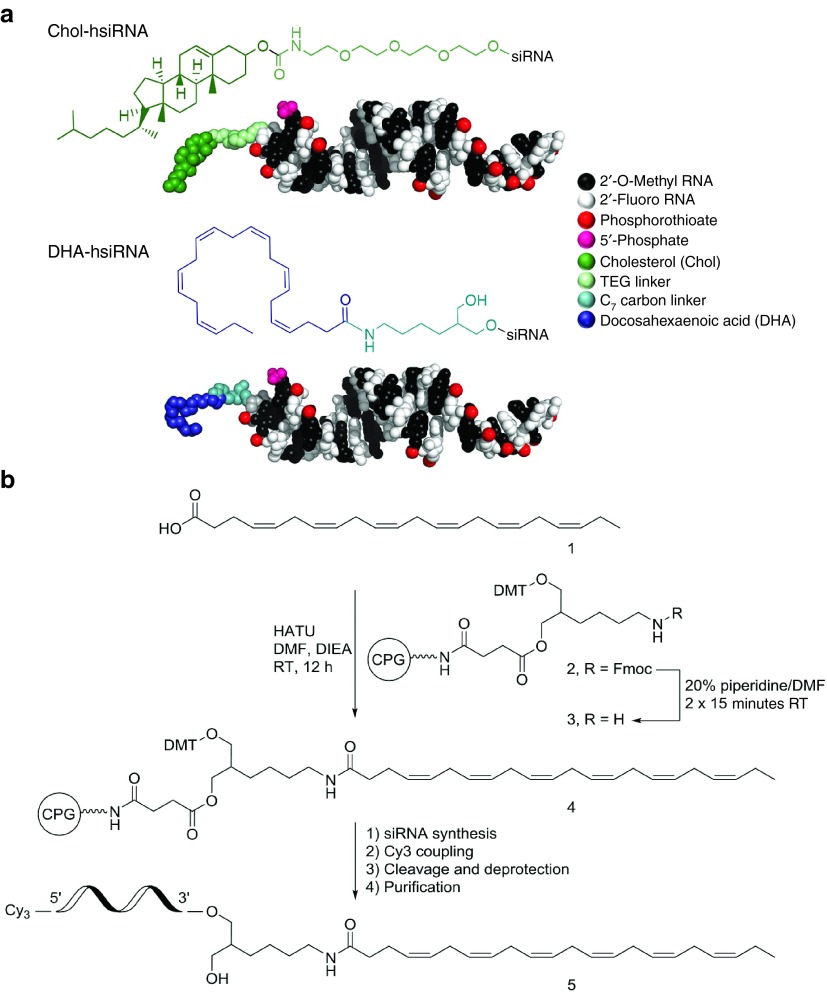
**Chemical structures and synthesis of hydrophobically modified siRNAs (hsiRNAs).** (**a**) Fully chemically stabilized hsiRNA with either docosahexaenoic acid (DHA) or cholesterol (Chol) bioconjugates at the 3′-end of the sense strand. Molecular models of hsiRNA represented to scale using PyMOL. The PyMOL Molecular Graphics System, Version 1.8 Schrödinger, LLC. (**b**) Solid-phase synthesis of DHA-conjugated hsiRNA. *Htt*, Huntingtin.

To evaluate the impact of DHA on siRNA distribution in the brain, we used a previously identified functional siRNA sequence targeting both human and mouse Huntingtin (*Htt*) mRNA.^11^ This compound is an asymmetric siRNA composed of a 20-nucleotide antisense (guide) strand and a 15-nucleotide sense (passenger) strand.^24^ This oligonucleotide is stabilized with alternating 2′-O-methyl and 2′-fluoro sugar modifications using a variant of a pattern first described by the Allerson *et al*.^25^ These extensive modifications are essential for the evaluation of conjugate-mediated delivery *in vivo,* as partially modified or unmodified siRNAs are rapidly degraded and cleared from the circulation and brain, limiting the ability to evaluate the conjugate's RNAi activity (Hassler *et al.*, 2016, manuscript submitted). The backbone of the terminal nucleotides is fully phosphorothioated to enhance stability against exonuclease-mediated degradation and to promote cellular internalization through a mechanism similar to that of antisense oligonucleotides (**Figure 1a**).^26^ The DHA moiety was conjugated through a commercially available C7 linker^27^ to the 3′-end of the sense strand via an amide bond (**Figure 1b**). Previous studies have identified the 3′-end of the sense strand to be an optimal position for conjugate attachment, with minimal effect on siRNA-RISC (intracellular RNA-induced silencing complex) loading.^28,29,30^ DHA-hsiRNA conjugates were synthesized on functionalized solid support bearing the DHA moiety (40 µmol/g) following standard solid-phase synthesis and deprotection protocols (see Materials and Methods). Newly synthesized oligonucleotides were purified by high-performance liquid chromatography and characterized by liquid chromatography–mass spectrometry (LC–MS) (**Supplementary Figure S1**). All oligonucleotide sequences and chemical modification patterns used in this study are presented in **Supplementary Table S1**.

### DHA-hsiRNA^*HTT*^ is internalized in primary cortical neurons and shows potent *Htt* mRNA silencing

For all siRNA studies, we applied the fully chemically modified scaffold described by Hassler *et al.* (2016, manuscript submitted) to a hyper-functional *Htt*-targeting siRNA sequence previously identified and validated in our lab (**Supplementary Table S1**).^11^

**Figure 2:**
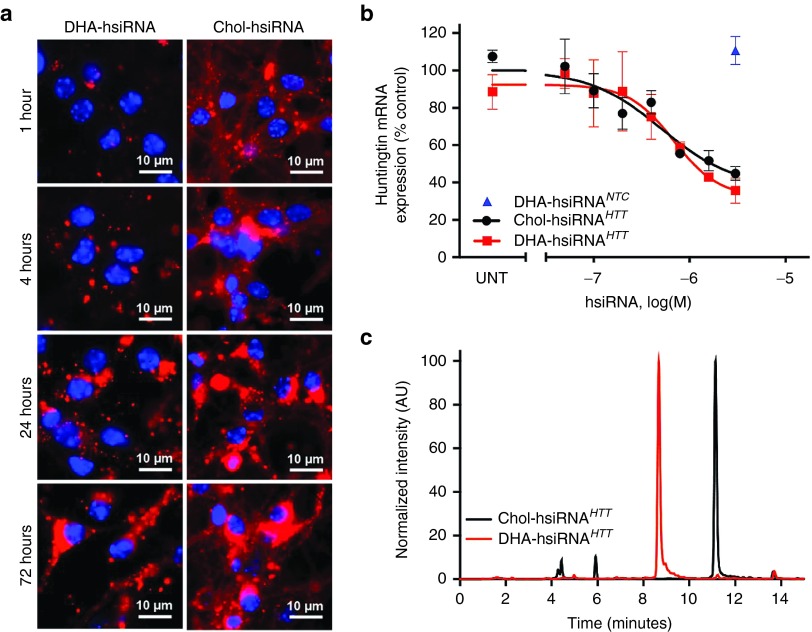
**DHA-hsiRNA shows slow neuronal uptake, equivalent Huntingtin (*Htt*) mRNA silencing, and reduced hydrophobicity compared with Chol-hsiRNA**. (**a**) Primary cortical neurons were incubated with Cy3-DHA-hsiRNA^*HTT*^ and Cy3-Chol-hsiRNA^*HTT*^ (0.5 µmol/l) for 0–72 hours. Images obtained with Leica inverted microscope (63×). Nuclei (Hoechst), blue; hsiRNA (Cy3), red. (**b**) Primary cortical neurons were incubated with Cy3-DHA-hsiRNA^*HTT*^ and Chol-hsiRNA^*HTT*^ at concentrations shown for 1 week. Level of huntingtin mRNA was measured using QuantiGene (Affymetrix) normalized to housekeeping gene, *Ppib* (cyclophillin B), and presented as percent of untreated control (*n* = 3, mean ± SD). UNT, untreated cells. (**c**) High-performance liquid chromatography (HPLC) traces of DHA-hsiRNA^*HTT*^ and Chol-hsiRNA^*HTT*^ following reverse phase chromatography (C8 column). *Htt*, Huntingtin; DHA, docosahexaenoic acid; DHA-hsiRNA, docosahexaenoic acid-hsiRNA conjugate; Chol-hsiRNA, cholesterol-hsiRNA conjugate.

We first analyzed and compared the live cell uptake kinetics of Chol-hsiRNA^*HTT*^ and DHA-hsiRNA^*HTT*^ in primary cortical neurons from wild-type (C57BL6) mice, using confocal imaging (**Figure 2a**). While hsiRNA^*HTT*^ rapidly associates with the cellular membrane (within minutes)^11^ and exhibits diffuse cytoplasmic staining, DHA-hsiRNA^*HTT*^ shows slower uptake kinetics to cytoplasmic foci with no detectable membrane binding. During early time points (of 4–6 hours) significant amounts of Chol-hsiRNA^*HTT*^ are detected inside the cells, while levels of internalized DHA-hsiRNA^*HTT*^ are minimal. Interestingly, the overall level of DHA-hsiRNA^*HTT*^ neuronal accumulation at 72 hours is comparable with that of Chol-hsiRNA^*HTT*^, resulting in similar levels of *Htt* silencing (**Figure 2b**). A secondary DHA-hsiRNA compound targeting cyclophilin B (*Ppib*) was evaluated to assess generality. After 1 week, DHA-hsiRNA^*PPIB*^ silences *Ppib* mRNA by 75% in mouse primary cortical neurons (**Supplementary Figure S2**). A DHA-conjugated hsiRNA containing a scrambled, nontargeting sequence had no effect on *Ppib* mRNA expression (**Supplementary Figure S2**). Nonconjugated hsiRNAs have modest efficacy *in vitro*, likely due to residual internalization by the single-stranded phosphorothioate tail, in a mechanism similar to that of conventional antisense oligonucleotides (**Supplementary Figure S3**). However, DHA conjugation results in enhancement of retention, distribution, and efficacy in mouse brain (vide infra).

To evaluate the impact of the bioconjugate on overall hsiRNA hydrophobicity, we compared the retention times of DHA-hsiRNA^*HTT*^ and Chol-hsiRNA^*HTT*^ using reverse phase chromatography (using a C8 modified column and triethylammonium acetate/acetonitrile eluents).^31,32^ We observed that DHA-hsiRNA^*HTT*^ eluted at 8.5 minutes while Chol-hsiRNA^*HTT*^ eluted at 11.8 minutes under these conditions, suggesting that DHA-hsiRNA^*HTT*^ is significantly less hydrophobic than cholesterol (**Figure 2c**, see Materials and Methods). This finding indicates that overall hsiRNA hydrophobicity can be strongly affected by the linked conjugate. While DHA- and Chol-hsiRNA^*HTT*^ conjugates have comparable activity in primary cortical neurons, we hypothesized that the reduction in overall compound hydrophobicity may improve pharmacokinetic properties *in vivo* in mouse brain.

### DHA-hsiRNA^*HTT*^ shows widespread distribution in the mouse brain following intrastriatal injection

**Figure 3:**
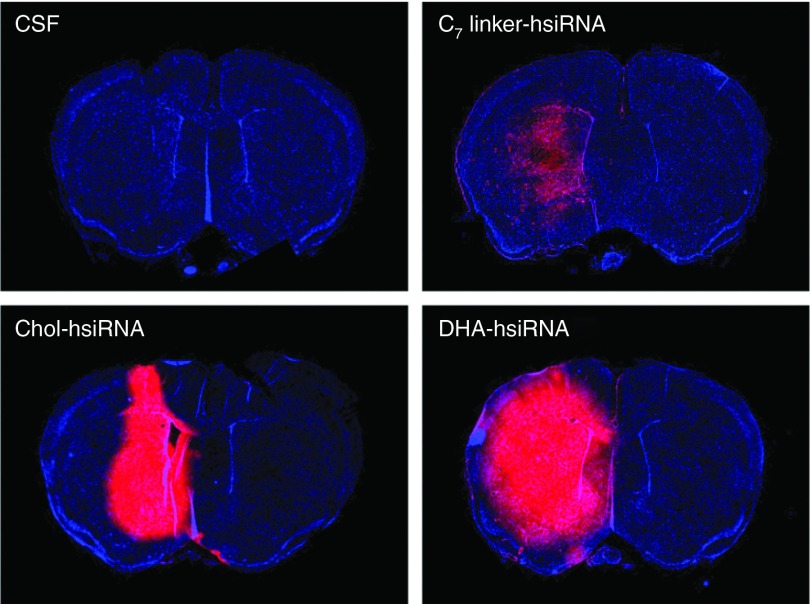
**DHA-hsiRNA^*HTT*^ shows widespread distribution in the mouse brain.** aCSF, Cy3-C_7_linker-hsiRNA^*HTT*^, Cy3-Chol-hsiRNA^*HTT*^, Cy3-DHA-hsiRNA^*HTT*^ were administered by unilateral intrastriatal injection (25 µg, 2 µl) and fluorescent images acquired by a Leica tiling microscope after 24 hours. Images consist of tiled-array of coronal sections at 10× magnification acquired with equivalent exposure and contrast settings. aCSF, artificial cerebrospinal fluid; Chol-hsiRNA, cholesterol-hsiRNA conjugate; DHA, docosahexaenoic acid; DHA-hsiRNA, docosahexaenoic acid-hsiRNA conjugate; *Htt*, Huntingtin.

**Figure 4:**
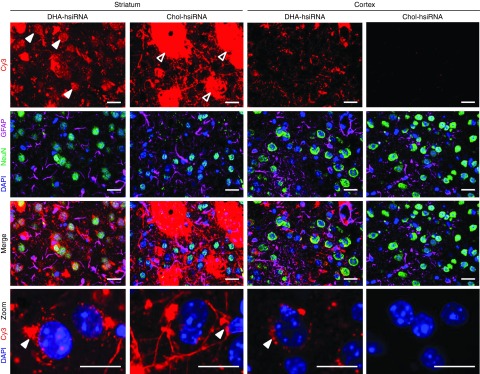
**DHA-hsiRNA exhibits clear neuronal localization in both the cortex and striatum following a unilateral intrastriatal injection.** Cy3-Chol-hsiRNA^*HTT*^ or Cy3-DHA-hsiRNA^*HTT*^ were administered by unilateral intrastriatal injection (25 µg, 2 µl) and fluorescent images acquired at 63× magnification by a Leica DMI6,000B microscope after 24 hours. Left two columns: ipsilateral striatum. Right two columns: ipsilateral cortex. Filled arrows indicate colocalization with nerve bundles. Unfilled arrows indicate subcellular localization to the perinuclear space. Enlarged inlets from merged image depicting neuronal uptake in the striatum (left) and cortex (right) for both Cy3-Chol-hsiRNA^*HTT*^ and Cy3-DHA-hsiRNA^*HTT*^ (bottom panels). Nuclei (DAPI), blue; NeuN, green; GFAP, magenta; Cy3-hsiRNA, red. Scale bar = 20 μm. DHA, docosahexaenoic acid; DHA-hsiRNA, docosahexaenoic acid-hsiRNA conjugate; *Htt*, Huntingtin.

Next, we evaluated the bio-distribution and neural cell uptake of Chol-hsiRNA^*HTT*^ and DHA-hsiRNA^*HTT*^ in mouse brain. When administered directly via a single intrastriatal injection, Cy3-labeled Chol-hsiRNA^*HTT*^ was primarily detected on the ipsilateral (injected) side of the brain (**Figure 3**). There is a steep gradient in distribution from the site of injection, however, with little detectable fluorescence present in the cortex or contralateral (noninjected) striatum (**Figure 3**). Chol-hsiRNA^*HTT*^ retention in the striatum may result from strong hydrophobic interactions with lipid-rich substructures (*e.g.* myelin-coated nerve bundles) in this region (**Figure 4**, unfilled arrows). Indeed, by high-resolution fluorescent microscopy (63X), we observe that Chol-hsiRNA^*HTT*^ mainly associates with hydrophobic myelin sheaths and appears to colocalize with striatal nerve bundles at the site of injection (**Figure 4**). Chol-hsiRNA^*HTT*^ is effectively internalized by neurons (**Figure 4**), and also, but to a smaller extent, by astrocytes (**Figure 4**). In neurons, Chol-hsiRNA^*HTT*^ is primarily observed in the neuronal processes, but also in the perinuclear area, the site of action of siRNAs (**Figure 4**, filled arrows).^33,34^

DHA-hsiRNA^*HTT*^ distributed more broadly than Chol-hsiRNA^*HTT*^ to both the ipsilateral striatum and cortex (**Figure 3**). This effect was specific to the DHA conjugate, as hsiRNA attached to the carbon linker alone was rapidly cleared (**Figure 3**). Although DHA-hsiRNA^*HTT*^ also colocalizes with striatal nerve bundles, the pattern of distribution and neuronal internalization significantly differs from Chol-hsiRNA^*HTT*^. In both striatal and cortical neurons, DHA-hsiRNA^*HTT*^ appears to primarily localize in the perinuclear area (**Figure 4**). Furthermore, the lower hydrophobicity of DHA-hsiRNA^*HTT*^ compared with Chol-hsiRNA^*HTT*^ appears to promote spread throughout the extracellular matrix and interstitial fluid, enabling an improved diffusion from the site of injection throughout the injected hemisphere.

### DHA-hsiRNA^*HTT*^ demonstrates significant, durable *Htt* mRNA silencing in both striatum and cortex following an intrastriatal injection

**Figure 5:**
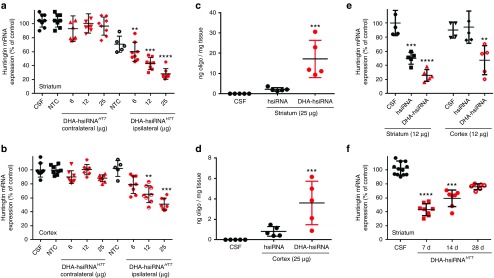
**DHA-hsiRNA^*HTT*^ efficacy and duration of effect following intrastriatal injection.** DHA-hsiRNA or nonconjugated hsiRNA were unilaterally injected into the striatum of wild-type mice. Punch biopsies of the striatum (**a**,**e**) and cortex (**b**,**e**) were collected after 5 days. For duration of effect **e**, punch biopsies of the striatum were collected at times shown. Level of *Htt* mRNA was measured using QuantiGene® (Affymetrix) normalized to housekeeping gene, *Ppib* (cyclophillin B), and presented as percentage of untreated control (*n* = 8 mice, mean ± SD). NTC, nontargeting control; aCSF, artificial cerebrospinal fluid (**d**,**e**) Plot showing tissue concentrations of DHA-hsiRNA^*HTT*^ guide strand measured by PNA hybridization from the ipsilateral striatum (**c**) or cortex (**d**). (*P < 0.05, ** *P* < 0.01, ***P < 0.001, ****P < 0.0001). DHA, docosahexaenoic acid; DHA-hsiRNA, docosahexaenoic acid-hsiRNA conjugate; PNA, peptide nucleic acid; *Htt*, Huntingtin.

Given the broad distribution of DHA-conjugated hsiRNAs in the brain, we wanted to evaluate its ability to induce gene silencing *in vivo*. Wild-type mice (FVB/NJ) were injected with artificial CSF, a nontargeting control hsiRNA (DHA-hsiRNA^NTC^, 25 µg), or DHA-hsiRNA^*HTT*^ (6–25 µg), into the right striatum (*n* = 8 per group). After 5 days, levels of *Htt* mRNA expression were measured by QuantiGene assay, normalized to a housekeeping gene (*Ppib*) and presented as percentage of an untreated control.^35^ We observed dose-dependent *Htt* silencing in both the striatum (up to 73%) and cortex (up to 51%) (**Figure 5a**,**b**). This degree of silencing in both cortex and striatum is consistent with the observed wide distribution pattern (**Figure 3**). While Chol-hsiRNA^*HTT*^ *Htt* silencing was equally as effective in the striatum, there was no statistically significant *Htt* knockdown observed in the cortex (data not shown). We observe similar levels of silencing of a secondary gene target using this chemistry platform with a Cyclophilin B-targeting DHA-hsiRNA (DHA-hsiRNA^*PPIB*^ **Supplementary Figure S4** and **Supplementary Figure S5**). A bilateral, 60 µg intrastriatal injection was performed and samples were collected for quantification of mRNA (QuantiGene) and protein expression. We observed robust *Ppib* silencing at both the mRNA and protein level, indicating that bilateral injection provides a simple and immediate ability to modulate gene expression in whole mouse striatum (**Supplementary Figure S4** and **Supplementary Figure S5**). We directly compared the levels of accumulation of a nonconjugated hsiRNA with a DHA-conjugated hsiRNA in both the striatum and cortex, using a quantitative peptide nucleic acid (PNA) hybridization assay.^36^ DHA improved retention in the striatum by ~10-fold, increasing retention from ~2 ng/mg tissue to ~18 ng/mg tissue (**Figure 5c**). DHA conjugation also improved retention in the cortex (~3.9 ng/mg tissue) compared with a nonconjugated hsiRNA, which accumulated to a lesser extent (~0.75 ng/mg tissue) (**Figure 5d**). This background retention of nonconjugated hsiRNA is likely a result of internalization mediated by the single-stranded phosphorothioated tail, described previously for antisense oligonucleotides. The level of retention correlated well with efficacy, as an unconjugated hsiRNA is only capable of achieving 50% *Htt* silencing in the striatum, with no detectable activity in the cortex following a single intrastriatal injection (12 µg, **Figure 5e**).

To evaluate the duration of effect, *Htt* silencing following a single, 12 µg DHA-hsiRNA^*HTT*^ injection was measured at 7, 14, and 28-day timepoints. The level of *Htt* silencing reduced over time, from ~60% at 7 days to 24% after 28 days. The 7 and 14-day timepoints were significant assuming a nonparametric distribution using a one-way analysis of variance with Dunns multiplicity correction (**Figure 5e**).

### DHA-hsiRNA^*HTT*^ does not induce measurable immune stimulation or adverse impact on neuronal viability over a broad dosage range

One of the major hurdles in the development of novel central nervous system-directed delivery technologies is dose-limiting toxicity. Typical lipid- and polymer-based siRNA formulations that improve pharmacokinetic properties when administered systemically exhibit pronounced toxicity and inflammatory response in the brain.^10,19^ To evaluate the safety of DHA-hsiRNA conjugates, we monitored changes in the expression of ionized calcium-binding adapter molecule 1 (IBA1) and dopamine- and cAMP-regulated neuronal phosphoprotein (DARPP-32), markers for innate immune stimulation and neuronal integrity, respectively. IBA-1 is a microglial-specific cell marker up-regulated following neuron injury, and IBA-1 staining is used to estimate levels of microglial activation following hsiRNA treatment by distinguishing between resting and activated microglia based on morphology.^37,38,39^ DARPP-32 is an established marker for striatal dopamine receptor activity and neuron viability.^40^

Previously, we have shown that partially modified Chol-hsiRNAs have no impact on DARPP-32 levels (neuronal viability) at efficacious levels, but induce a slight increase in the level of activated microglia using an IBA-1 marker.^11^ When cholesterol was conjugated to fully modified scaffold utilized herein, severe toxicity was observed at doses higher than 25 µg, causing mortality in ~30% of injected animals (data not shown). This pronounced increase in toxicity is attributed to poor distribution from the site of injection, with excess accumulation of the hydrophobic, chemically stabilized hsiRNA causing neuronal loss, consistent with the hypothesis that a high local compound concentration is toxic within brain tissues.

**Figure 6:**
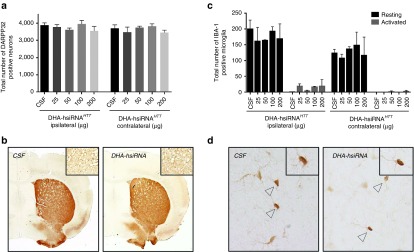
**DHA-hsiRNA^*HTT*^ shows no impact on neuronal integrity or innate immune activation at ~20-fold higher concentrations than what is required for activity.** DHA-hsiRNA^*HTT*^ was administered by unilateral intrastriatal injection. Brains were collected after 5 days, fixed, sectioned, and stained with antibodies against DARPP-32, a marker for spiny medium neurons, or IBA-1, a marker for microglia. (**a**) Data represented as total number of DARPP-32 positive neurons per tissue section (*n* = 3 mice, mean ± SD). (**b**) Representative images of DARPP-32 staining. (**c**) Data represented as total number of activated microglia (*n* = 3 mice, mean ± SD). (**d**) Representative images of IBA-1 staining. IBA1, ionized calcium-binding adapter molecule 1 DHA, docosahexaenoic acid; DHA-hsiRNA, docosahexaenoic acid-hsiRNA conjugate; PNA, peptide nucleic acid; *Htt*, Huntingtin.

To evaluate the toxicity of DHA-hsiRNA *in vivo*, animals were injected with a broad range of DHA-hsiRNA concentrations (25–200 µg). Given the solubility limit of DHA-hsiRNA (10 mmol/l in artificial CSF (aCSF)) and the injection volume (2 µl), 200 µg is the highest possible dose that can be administered intrastriatally, and 25 µg is fourfold higher than what is required for detectible silencing activity (**Figure 5**). We observed no reduction in DARPP-32 levels (**Figure 6a**) or significant elevation of activated microglia (**Figure 6b**) in coronal brain sections of mice treated at the highest dose level. Moreover, all injected animals appeared normal, with no signs of distress or toxicity. These results indicate that administration of DHA-hsiRNA has no measureable impact on neuronal integrity or innate immune system activation at the dose levels tested (**Figure 6**).

## Discussion

Direct conjugation of siRNA to small, bioactive ligands has the potential to allow efficient, targeted tissue delivery, which remains an outstanding challenge in the field of therapeutic RNAi. This strategy is particularly promising for brain applications, where conventional siRNA carriers (including lipid-based formulations, polymers, nanoparticles, or dendrimers) are not generally well tolerated.^8,9,10^ Here, we report that direct conjugation of DHA to a fully chemically stabilized siRNA scaffold shows significant tissue retention with wide distribution and robust efficacy in mouse brain. Notably, DHA-hsiRNA conjugates do not elicit measurable microglial activation and have no adverse effect on neuronal viability at concentrations over 20-fold higher than the efficacious dose.

DHA-hsiRNA alleviates one of the major obstacles to neurological applications of siRNA, which is achieving widespread distribution to brain parenchyma. Following a direct intrastriatal injection, DHA-hsiRNA distributed broadly throughout the striatum and cortex of the injected hemisphere, with no dramatic compound accumulation around the site of injection (a typical feature of Chol-hsiRNA). We observed DHA-hsiRNA colocalization with both neuronal (neuronal nuclei antigen (NeuN)) and astrocyte (glial fibrillary acidic protein (GFAP)) markers. DHA-hsiRNA clearly localized to the perinuclear space in both striatal and cortical neurons (the cytoplasmic site of active RNAi).

DHA-hsiRNA accumulates to a functional degree in both the striatum and cortex after a single, intrastriatal injection. *Htt* silencing is achieved at concentrations as low as 6 µg (~25% silencing) in the striatum and 12 µg (~30% silencing) in the cortex. A maximal knockdown of 70–80% was seen following administration of 25 µg in the striatum. Duration of effect studies reveal persistent *Htt* mRNA silencing in mouse striatum up to 4 weeks after a single, 12 µg intrastriatal injection. We observed comparable levels of gene silencing of a secondary target, *Ppib*, with the DHA-hsiRNA chemistry platform. We utilized a bilateral intrastriatal injection of DHA-hsiRNA^*PPIB*^ to quantify the reduction in *Ppib* mRNA and protein levels throughout both hemispheres, achieving up to ~80% silencing in the striatum. These results suggest that DHA-hsiRNA has immediate potential to modulate gene expression in animal models of neurodegenerative disorders.

We were encouraged by the results of the pilot safety study. Comparing increasing concentrations of fully chemically modified DHA-hsiRNA and Chol-hsiRNA, we found that Chol-hsiRNA induces significant loss of brain matter and occasionally animal morbidity at doses above 25 µg (data not shown). In contrast, animals injected with 200 µg of DHA-hsiRNA appear healthy, with normal brain morphology. 200 µg is the maximal amount that can be delivered intrastriatally, given the solubility limit of DHA-hsiRNA. The acute toxicity observed following Chol-hsiRNA administration is likely due to elevated compound retention around the site of injection, as a less stable, partially modified Chol-hsiRNA has a minimal effect on neuronal viability, following direct intrastriatal injection.^11^ The reduced hydrophobicity and broad distribution of fully chemically modified DHA-hsiRNA precludes accumulation around the site of injection, and likely supports its high tolerance and safety *in vivo*. This chemistry may support an even wider therapeutic index following other clinically feasible routes of administration (*e.g.*, intrathecal, intracerebroventricular, or systemic intravenous), administration of higher doses, or through multiple-dose regimens. For instance, antisense oligonucleotides delivered through a bolus intrathecal administration in rodents are dosed at up to 600 µg total.^41^ A long-term safety study, including measurement of cytokine levels to further assess immunogenicity, as well as aspartate transaminase (AST)/ alanine transaminase (ALT) blood ratios to determine hepatotoxicity, would corroborate the results presented in this study.

The mechanism behind the safety profile of DHA-hsiRNA is unknown. A comprehensive understanding of the DHA-hsiRNA safety profile is essential for clinical development of these compounds for therapeutic applications. DHA is highly abundant in the mammalian brain, of which DHA and related polyunsaturated fatty acids comprise >50% of neural cell phospholipid membranes. DHA metabolites are involved in signal transduction, cell survival, and alleviating neuroinflammation.^15^ It is possible that the DHA moieties from DHA-hsiRNA conjugates may enter into endogenous fatty acid trafficking, phospholipid biosynthesis, and metabolic pathways of neural cells, contributing to tissue health and overall compound safety.^42,43,44^

DHA is a single representative of a class of biologically active polyunsaturated fatty acids. While saturated or monounsaturated lipids have been extensively explored for siRNA delivery,^45,46,47,48^ polyunsaturated fatty acid-siRNA conjugates are less well described. It would be interesting to investigate the tissue distribution and efficacy of other polyunsaturated fatty acid-hsiRNA conjugates, such as eicosapentaenoic acid. It would also be intriguing to determine whether these types of conjugates can enhance the efficacy of other classes of therapeutic oligonucleotides, like antisense or splice-switching oligonucleotides. We believe that it is likely, as long the oligonucleotide modality is chemically stabilized sufficiently to be stable in tissue for extended periods of time.

In this study, we targeted *Htt*, the causative gene of Huntington's disease. Currently prescribed small molecule drugs for genetically defined neurodegenerative diseases, such as Huntington's disease, seek to treat disease symptoms without addressing the underlying genetic cause. A major advantage of RNAi-based therapeutics is that it permits specific targeting of the gene(s) underlining the clinical pathology. It has been shown that transient modulation of both wild-type and mutant *Htt* alleles were sufficient to support reversal of disease phenotype. An antisense compound targeting both alleles has recently advanced into clinical trials.^41^ DHA-hsiRNA^*HTT*^ demonstrates robust and durable silencing in both striatum and cortex, the brain regions primarily affected in Huntington's disease. The data presented here describe a promising novel platform for modulation of gene expression *in vivo* in the brain, paving the path toward better therapeutics for the treatment of dominant and hereditable neurodegenerative diseases including Huntington's disease.

## Materials and methods

*Materials.* DHA was from MP Biomedicals (Solon, OH) and 1-O-DMT-6-N−Fmoc-2-hydroxymethylhexane from Toronto Research Chemicals (Ontario, Canada). RNA synthesis reagents and controlled pore glass functionalized with long chain alkyl amine (LCAA-CPG) were obtained from ChemGenes (Wilmington, MA). Quasar 570 CE phosphoramidite (Cy3) was purchased from Gene Pharma (Shanghai, China).

*Preparation of amine-bearing CPG.* A functionalized CPG (**3**, **Figure 1**) was prepared and used for the solid-phase conjugation of DHA. First, LCAA-CPG (particle size 125–177 µm, pore diameter 500 Å and primary amino loading 145 µmol/g) was activated and dried over night according to published protocols.^49^ Then, the commercially available 1-O-DMT-6-N−Fmoc-2-hydroxymethylhexane was converted to succinate and loaded on CPG following a reported procedure to afford **2**.^50^ The linker loading was determined by DMT assay to be around 50 µmol/g. Subsequently, the Fmoc group was removed from **2** using a solution of 20% piperidine in dimethylformamide for 15 minutes. This procedure was repeated twice to ensure complete deprotection of the Fmoc group. The amine-bearing CPG **3** was filtered off and washed successively with CH_2_CI_2_, ACN, and ether then dried under vacuum.

*DHA coupling.* 38 mg of DHA (40.3 µl, 115.6 µmol) was transferred to a 5 ml flask and dissolved in 3 ml of dry dimethyl formamide (DMF). The solution was degassed and kept under argon. Then 11.4 mg (30 µmol) of 1-[Bis(dimethylamino)methylene]-1H-1,2,3-triazolo[4,5-b]pyridinium 3-oxid hexafluorophosphate (HATU) was added to the flask and the solution was stirred for 3 minutes. Five eq. of Diisopropylethylamine (DIEA) was added to the solution followed by 600 mg (30 µmol, loading 50 µmol/g) of amine-bearing CPG **3**. The flask was sealed and rocked gently at room temperature. After 12 hours, the CPG was filtered off and washed successively with CH_2_CI_2_, acetonitrile (ACN), and ether, then dried under vacuum.

*Synthesis of oligonucleotides.* Oligonucleotides were synthesized on an Expedite ABI DNA/RNA Synthesizer following standard protocols. Each synthesis was done at a 1-µmole scale using DHA-conjugated CPG for the sense (see above) and a Unylinker terminus (ChemGenes) for the antisense strands. Phosphoramidites were prepared as 0.15 mol/l solutions for 2′-O-methyl (ChemGenes) and Cy3 (Gene Pharma) and 0.13 mol/l for 2′-fluoro (BioAutomation, Irving, Texas) in ACN. 5-(Ethylthio)-1H-tetrazole (ETT) 0.25 mol/l in ACN was used as coupling activator. Detritylations were performed using 3% dichloroacetic acid in CH_2_Cl_2_ for 80 seconds and capping was done with a 16% N-methylimidazole in THF (CAP A) and acetic anhydride:pyridine:THF, (1:2:2, v/v/v) (CAP B) for 15 seconds. Sulfurizations were carried out with 0.1 mol/l solution of ((dimethylamino-methylidene)amino)-H-1,2,4-dithiazaoline-3-thione (DDTT) in ACN for 3 minutes. Oxidation was performed using 0.1 mol/l iodine in pyridine:water:THF (1:2:10, v/v/v). Phosphoramidite coupling times were 350 seconds for all amidites.

*Deprotection and purification of oligonucleotides.* Both sense and antisense strands were cleaved and deprotected using 1 ml of 40% aq. methylamine at 65°C for 10 minutes. The oligonucleotide solutions were then cooled in a freezer for a few minutes and dried under vacuum in a Speedvac. The resulting pellets were suspended in 10 ml of triethylammonium acetate buffer (0.1 mol/l, pH 7) and filtered through a 0.2 µm filter. The final purification of oligonucleotides was performed on an Agilent Prostar System (Agilent, Santa Clara, CA) equipped with a Hamilton HxSil C8 column (150 × 21.2) using the following conditions: buffer A: (0.1 mol/l, TEAA, PH 7), B: (ACN), gradient: 10% B, 90% A to 90% B, 10% A in 30 minutes, temperature: 55°C, flow rate: 20 ml/minutes. The pure oligonucleotides were collected and cation-exchanged on a HiTrap 5 ml SP HP column (GE Healthcare Life Sciences, Marlborough, MA) and lyophilized. The overall yields of DHA-conjugated siRNAs were constantly above 60%.

*LC–MS analysis of oligonucleotides.* The identity of oligonucleotides were established by LC–MS analysis on a Waters Q-TOF premier machine (Waters Corporation, Milford, MA) using following conditions: buffer A (15 mmol/l dibutylamine/25 mmol/l hexafluoroisopropanol), buffer B (20% A in MeOH), column: xbidge OST C18 (Waters Corporation), 2.5µm, gradient: 0–10 minutes (1% B-80% B), 10–13 minutes (80% B-80% B), 13.1 minutes (80% B-1% B), 13.1–18 minutes (1% B-1% B). A sample LC–MS chromatogram of the DHA-conjugated siRNAs is shown in **Supplementary Figure S1**.

*Preparation of primary neurons.* Primary cortical neurons were isolated and cultured from FVB/NJ mouse embryos at embryonic day 15.5 as described previously.^11^ Briefly, the cortices from both embryonic brain hemispheres were dissected, and a solution of papain and DNAse I (Worthington, Lakewood, NJ) in Hibernate E medium (Brainbits, Springfield, IL) was used to disrupt the extracellular matrix prior to trituration to a single-cell suspension using a fire-polished glass pipette. Cortical neurons were plated at 1 × 10^5^ cells/well in a poly-L-lysine coated 96-well plate (Sigma, St. Louis, MO) in 100 µl of NBActiv4 (Brainbits) supplemented with 1.5% fetal bovine serum (Sigma). The following day, the cellular media was supplemented with antimitotics to prevent growth of nonneuronal cells (0.48 µl/ml UTP and 0.2402 µ/ml of FdUMP, Sigma). Neurons were treated with oligonucleotides on day 3 after plating.

*Direct delivery (passive uptake) of oligonucleotides.* Cells were plated in Dulbecco's modified essential medium containing 6% fetal bovine serum at 10,000 cells per well in 96-well tissue culture plates. hsiRNA was diluted to twice the final concentration in OptiMEM (Gibco, Carlsbad, CA), and 50 μl diluted hsiRNA was added to 50 μl of cells, resulting in 3% fetal bovine serum final. Cells were incubated for 72 hours at 37°C and 5% CO_2_.

*Confocal imaging.* All confocal images were acquired with a CSU10B Spinning Disk Confocal System scan head (Solamere Technology Group) mounted on a TE-200E2 inverted microscope (Nikon, Melville, NY) with a 60x Plan/APO oil lens and a Coolsnap HQ2 camera (Roper, Acton, MA). Images were processed using ImageJ (1.47v) software.

*mRNA quantification in cells and tissue punches.* mRNA was quantified from both cells and tissue punches using the QuantiGene 2.0 Branched DNA (bDNA) Assay (Affymetrix, Santa Clara, CA) as described previously.^35^ Probe sets (Mouse *Htt* or *Ppib*, #SB-14,150 and #SB-10,002) were diluted and used according to the manufacturer's recommended protocol. Briefly, tissue punches (5 mg) were homogenized in 300 μl of Homogenizing Buffer (Affymetrix) containing 2 μg/μl proteinase K in 96-well plate format on a QIAGEN TissueLyser II and 40 μl of each lysate was added to a bDNA capture plate. *Htt* or *Ppib* probe sets (60 µl) were added to each well of the capture plate for a final volume of 100 μl. Signal was amplified according to the Affymetrix protocol. Luminescence was detected on a Tecan M1,000 luminometer.

*Live cell imaging.* To monitor live cell DHA- and Chol-hsiRNA uptake, primary cortical neurons were plated at a density of 2 × 10^5^ cells per poly-L-lysine coated, 35 mm glass-bottom dish. Cell nuclei were stained with NucBlue (Life Technologies, Carlsbad, CA) according to the manufacturer's recommended protocol. Imaging was performed in phenol red-free NbActiv4 medium (Brainbits). Cells were treated with 0.5 μmol/l Cy3-labeled hsiRNA, and live cell imaging was performed over time. All live cell confocal images were acquired with a Zeiss confocal microscope, and images were processed using ImageJ (1.47v) software as described previously.

*Stereotaxic injections and tissue collection.* FVB/NJ male and female mice (6–8 weeks old) were deeply anesthetized with 1.2% Avertin (Sigma) and microinjected by stereotactic placement into the right striatum (coordinates relative to bregma: 1.0 mm anterior, 2.0 mm lateral, and 3.0 mm ventral).

For efficacy studies, animals were injected unilaterally or bilaterally with 2 μl of artificial CSF (aCSF), DHA-hsiRNA^*NTC*^ (12.5 μg), and DHA-hsiRNA^*HTT*^ (3.1, 6.3, 12.5, 25, or 60 µg) or the doses indicated for the toxicity studies. For efficacy studies *n* = 8 mice were injected per group, and for toxicity studies *n* = 3 mice were injected per group. Mice were euthanized 5 days postinjection and brains harvested. For efficacy studies, three 300 μm coronal sections were collected and from each section, a 2 mm punch was taken from each side (injected and noninjected) and placed in RNA later (Ambion, Waltham, MA) for 24 hours at 4°C. Each punch was processed as an individual sample for QuantiGene 2.0 assay analysis (Affymetrix), and averaged for a single animal point. For DARPP-32 assessment toxicity studies, animals were perfused with phosphate-buffered saline and 10% formalin solution, and postfixed for 24 hours. For microglial activation studies, brains were harvested after 6 hours or 5 days. Brains were sliced into 40 µm sections on the Leica 2,000T Vibratome (Leica Biosystems, Buffalo Grove, IL) in ice cold PBS, and stored at 4ºC until immunostaining was carried out.

For biodistribution studies, mice were injected into the right striatum with 2 μl of aCSF, Cy3-Chol-hsiRNA^*HTT*^ (25 μg) or Cy3-DHA-hsiRNA^*HTT*^ (25 μg). After 48 hours, mice were euthanized and perfused with PBS and 10% formalin solution, and brains postfixed for 48 hours. Afterwards, brains were paraffin-embedded and sliced into 4 μm sections that were mounted on glass slides. All animal procedures were approved by the University of Massachusetts Medical School Institutional Animal Care and Use Committee (IACUC, protocol number A-2411).

*PNA hybridization assay.* Overall levels of hsiRNA guide (antisense) strand in mouse brain were quantified using a modified PNA hybridization assay. Tissue samples from the efficacy studies were lysed in MasterPure Tissue Lysis Solution (EpiCentre) in the presence of proteinase K (2 mg/ml) (Invitrogen, #25530-049) and homogenized using a TissueLyser II (Qiagen), with roughly 10 mg tissue per 100 μl lysis solution. Sodium dodecyl sulfate from lysate was precipitated with KCl (3 mol/l) and pelleted at 5,000 g. hsiRNA present in the cleared supernatant was hybridized to a Cy3-labeled PNA fully complementary to guide strand (PNABio, Thousand Oaks, CA) and injected into high-performance liquid chromatography DNAPac PA100 anion exchange column (Thermo Fisher Scientific). Cy3 fluorescence was monitored and peaks integrated. Mobile phase for high-performance liquid chromatography was 50% water, 50% acetonitrile, 25 mmol/l Tris-HCl (pH 8.5), and 1 mmol/l ethylenediaminetetraacetate. The salt gradient was 0–800 mmol/l NaClO_4_. For an internal calibration curve, a known amount of hsiRNA duplex was spiked into the tissue lysis solution.

*Immunohistochemistry and immunofluorescence.* For biodistribution studies, tissue sections on glass slides were deparaffinized by incubating samples twice for 8 minutes in Xylene. Sections were rehydrated by incubating samples in a series of ethanol dilutions (100, 95, and 80%), for 4 minutes each. Slides where then washed twice for 2 minutes with PBS.

NeuN and GFAP immunostaining was carried out after antigen retrieval by boiling tissue sections for 5 minutes in 10 mmol/l Tris/ 1mmol/l ethylenediaminetetraacetate (pH 9.0) buffer, followed by 20 minutes incubation at room temperature. Sections were then washed for 5 minutes in PBS and blocked for 1 hour in 5% normal goat serum in PBS containing 0.05% Tween 20 (PBST). Slides were washed for 5 minutes with PBST, and incubated with anti-mouse NeuN (1:1,000 dilution) (Millipore, Billerica, MA) or anti-mouse GFAP (1:340) (Dako, Santa Clara, CA) primary antibody in PBST. Sections were washed three times with PBST for 5 minutes, and incubated with respective secondary antibody for 30 minutes in the dark. Secondary antibodies used were antimouse Alexa 488 (1:1,000) (Life Technologies) and anti-rabbit Alexa 647 (1:1,000 dilution) (Life Technologies). Slides were washed three times with PBST for 5 minutes and counterstained with 2-(4-amidinophenyl)-1H-indole-6-carboxamidine (DAPI) (250 ng/ml) (Molecular Probes, Life Technologies) in PBS for 2 minutes followed by a wash in PBS for 2 minutes. Slides were mounted with mounting medium and coverslips and dried overnight in the cold room. Tiled-arrays of coronal sections were acquired on a Leica DM5500B microscope and high-resolution images at 63× acquired on a Leica DMI6000 microscope, both microscopes were fitted with a DFC365 FX fluorescence camera.

For toxicity studies, 40 μm formalin-fixed sections were assessed for DARPP-32 and/or IBA1 immunostaining. A total of 9 sections per brain and 8 images per section (4 per hemisphere) were evaluated. Sections for IBA-1 immunostaining were incubated in blocking solution (5% normal goat serum, 1% bovine serum albumin, 0.2% Triton-X-100, and 0.03% hydrogen peroxide in PBS) for 1 hour, and then washed with PBS. Later, sections were incubated overnight at 4ºC in primary antibody, anti-Iba1 (1:500 dilution in blocking solution) (Wako, Richmond, VA). Sections were then stained with goat antirabbit secondary antibody (1:200 dilution) (Vector Laboratories, Burlingame, CA) for 1 hour. Slides were washed with PBS, and incubated with the Vectastain ABC Kit (Vector Laboratories), followed by another PBS wash. IBA-1 was detected with the Metal Enhanced DAB Substrate Kit (Pierce, Dallas, TX). For DARPP32 staining, sections were incubated for 3 minutes in 3% hydrogen peroxide, followed by 20 minutes in 0.2% Triton-X-100. Samples were incubated in 1.5% normal goat serum in PBS for 4 hours, and thereafter incubated overnight at 4ºC in DARPP32 primary antibody (1:10,000 dilution in 1.5% normal goat serum) (Abcam, Cambridge, UK). Secondary antibody and detection steps were conducted as described for IBA-1 staining. Sections were mounted and cover-slipped. A Nikon Eclipse E600 microscope with a Nikon Digital Sight DSRi1 camera was used to visualize and acquire bright field images at 20x or 40x for counting and analysis. The number of DARPP-32-positive neurons was quantified manually using the cell counter plug-in on ImageJ for tracking and activated microglia was quantified by morphology of IBA1-positive cells. All sections were coded and randomized to enable blinded counting of both IBA1 and DARPP-32 positive cells. Coronal section images were acquired using a Coolscan V-ED LS50 35 mm Film Scanner (Nikon).

*Statistical analysis.* Data were analyzed using GraphPad Prism 6 software (GraphPad Software, San Diego, CA). Concentration-dependent IC50 curves were fitted using a log(inhibitor) versus response – variable slope (four parameters). The lower limit of the curve was set at zero, and the upper limit of the curve was set at 100. For each independent mouse experiment, the level of knockdown at each dose was normalized to the mean of the control group (the noninjected side of the PBS or artificial CSF groups). *In vivo* data were analyzed using nonparametric Kruskal-Wallis test of one-way analysis of variance with Dunn's multiple comparisons test. Differences in all comparisons were considered significant at *P*-values ≤ 0.05 compared with the noninjected (contralateral) side of striatum for the NTC injected group. For microglial activation, significance was calculated using a parametric, unpaired, two-tailed *t*-test for comparison between dose groups.

**SUPPLEMENTARY MATERIAL** **Figure S1.** Representative LC-MS characterization of Cy3-DHA-hsiRNAHTT. **Figure S2.** DHA-hsiRNAPPIB silences Cyclophilin B (Ppib) mRNA in mouse primary cortical neurons. **Figure S3.** Non-conjugated hsiRNAHTT exhibits modest Huntingtin (Htt) mRNA silencing in mouse primary cortical neurons. **Figure S4.** DHA-hsiRNAPPIB silences Cyclophilin B (Ppib) mRNA in mouse striatum following a single intrastriatal injection. **Figure S5.** DHA-hsiRNAPPIB silences Cyclophilin B (PPIB) protein in mouse striatum following a single intrastriatal injection. **Table S1**. Modified Oligonucleotide Sequences.

## References

[bib1] Mathupala, SP (2009). Delivery of small-interfering RNA (siRNA) to the brain. Expert Opin Ther Pat 19: 137–140. 19441914 10.1517/13543770802680195 PMC2698461

[bib2] Zukiel, R, Nowak, S, Wyszko, E, Rolle, K, Gawronska, I, Barciszewska, MZ et al. (2006). Suppression of human brain tumor with interference RNA specific for tenascin-C. Cancer Biol Ther 5: 1002–1007. 16775434 10.4161/cbt.5.8.2886

[bib3] Zhang, Y, Zhang, YF, Bryant, J, Charles, A, Boado, RJ and Pardridge, WM (2004). Intravenous RNA interference gene therapy targeting the human epidermal growth factor receptor prolongs survival in intracranial brain cancer. Clin Cancer Res 10: 3667–3677. 15173073 10.1158/1078-0432.CCR-03-0740

[bib4] Grondin, R, Ge, P, Chen, Q, Sutherland, JE, Zhang, Z, Gash, DM et al. (2015). Onset time and durability of huntingtin suppression in Rhesus putamen after direct infusion of antihuntingtin siRNA. Mol Ther Nucleic Acids 4: e245. 26125484 10.1038/mtna.2015.20 PMC4561652

[bib5] Shepherd, GM (2013). Corticostriatal connectivity and its role in disease. Nat Rev Neurosci 14: 278–291. 23511908 10.1038/nrn3469 PMC4096337

[bib6] Wang, H, Ghosh, A, Baigude, H, Yang, CS, Qiu, L, Xia, X et al. (2008). Therapeutic gene silencing delivered by a chemically modified small interfering RNA against mutant SOD1 slows amyotrophic lateral sclerosis progression. J Biol Chem 283: 15845–15852. 18367449 10.1074/jbc.M800834200 PMC2414310

[bib7] DiFiglia, M, Sena-Esteves, M, Chase, K, Sapp, E, Pfister, E, Sass, M et al. (2007). Therapeutic silencing of mutant huntingtin with siRNA attenuates striatal and cortical neuropathology and behavioral deficits. Proc Natl Acad Sci USA 104: 17204–17209. 17940007 10.1073/pnas.0708285104 PMC2040405

[bib8] Grimm, D (2011). The dose can make the poison: lessons learned from adverse *in vivo* toxicities caused by RNAi overexpression. Silence 2: 8. 22029761 10.1186/1758-907X-2-8 PMC3234190

[bib9] Krichevsky, AM and Kosik, KS (2002). RNAi functions in cultured mammalian neurons. Proc Natl Acad Sci USA 99: 11926–11929. 12192088 10.1073/pnas.182272699 PMC129370

[bib10] Godinho, BM, McCarthy, DJ, Torres-Fuentes, C, Beltrán, CJ, McCarthy, J, Quinlan, A et al. (2014). Differential nanotoxicological and neuroinflammatory liabilities of non-viral vectors for RNA interference in the central nervous system. Biomaterials 35: 489–499. 24138827 10.1016/j.biomaterials.2013.09.068

[bib11] Alterman, JF, Hall, LM, Coles, AH, Hassler, MR, Didiot, MC, Chase, K et al. (2015). Hydrophobically modified siRNAs silence huntingtin mRNA in primary neurons and mouse brain. Mol Ther Nucleic Acids 4: e266. 26623938 10.1038/mtna.2015.38 PMC5014532

[bib12] Dyall, SC (2015). Long-chain omega-3 fatty acids and the brain: a review of the independent and shared effects of EPA, DPA and DHA. Front Aging Neurosci 7: 52. 25954194 10.3389/fnagi.2015.00052 PMC4404917

[bib13] Glomset, JA (2006). Role of docosahexaenoic acid in neuronal plasma membranes. Sci STKE 2006: pe6. 16467193 10.1126/stke.3212006pe6

[bib14] Salem, N Jr, Litman, B, Kim, HY and Gawrisch, K (2001). Mechanisms of action of docosahexaenoic acid in the nervous system. Lipids 36: 945–959. 11724467 10.1007/s11745-001-0805-6

[bib15] Bazinet, RP and Layé, S (2014). Polyunsaturated fatty acids and their metabolites in brain function and disease. Nat Rev Neurosci 15: 771–785. 25387473 10.1038/nrn3820

[bib16] Serhan, CN, Chiang, N and Van Dyke, TE (2008). Resolving inflammation: dual anti-inflammatory and pro-resolution lipid mediators. Nat Rev Immunol 8: 349–361. 18437155 10.1038/nri2294 PMC2744593

[bib17] Kohli, P and Levy, BD (2009). Resolvins and protectins: mediating solutions to inflammation. Br J Pharmacol 158: 960–971. 19594757 10.1111/j.1476-5381.2009.00290.x PMC2785519

[bib18] Calder, PC (2013). Omega-3 polyunsaturated fatty acids and inflammatory processes: nutrition or pharmacology? Br J Clin Pharmacol 75: 645–662. 22765297 10.1111/j.1365-2125.2012.04374.x PMC3575932

[bib19] Whitehead, KA, Dahlman, JE, Langer, RS and Anderson, DG (2011). Silencing or stimulation? siRNA delivery and the immune system. Annu Rev Chem Biomol Eng 2: 77–96. 22432611 10.1146/annurev-chembioeng-061010-114133

[bib20] Merendino, N, Costantini, L, Manzi, L, Molinari, R, D'Eliseo, D and Velotti, F (2013). Dietary ω-3 polyunsaturated fatty acid DHA: a potential adjuvant in the treatment of cancer. Biomed Res Int 2013: 310186. 23762838 10.1155/2013/310186 PMC3676987

[bib21] Siddiqui, RA, Harvey, KA, Xu, Z, Natarajan, SK and Davisson, VJ (2014). Characterization of lovastatin-docosahexaenoate anticancer properties against breast cancer cells. Bioorg Med Chem 22: 1899–1908. 24556504 10.1016/j.bmc.2014.01.051

[bib22] Willibald, J, Harder, J, Sparrer, K, Conzelmann, KK and Carell, T (2012). Click-modified anandamide siRNA enables delivery and gene silencing in neuronal and immune cells. J Am Chem Soc 134: 12330–12333. 22812910 10.1021/ja303251f

[bib23] Felber, AE, Bayó-Puxan, N, Deleavey, GF, Castagner, B, Damha, MJ and Leroux, JC (2012). The interactions of amphiphilic antisense oligonucleotides with serum proteins and their effects on *in vitro* silencing activity. Biomaterials 33: 5955–5965. 22656448 10.1016/j.biomaterials.2012.05.019

[bib24] Byrne, M, Tzekov, R, Wang, Y, Rodgers, A, Cardia, J, Ford, G et al. (2013). Novel hydrophobically modified asymmetric RNAi compounds (sd-rxRNA) demonstrate robust efficacy in the eye. J Ocul Pharmacol Ther 29: 855–864. 24180627 10.1089/jop.2013.0148

[bib25] Allerson, CR, Sioufi, N, Jarres, R, Prakash, TP, Naik, N, Berdeja, A et al. (2005). Fully 2'-modified oligonucleotide duplexes with improved *in vitro* potency and stability compared to unmodified small interfering RNA. J Med Chem 48: 901–904. 15715458 10.1021/jm049167j

[bib26] Geary, RS, Norris, D, Yu, R and Bennett, CF (2015). Pharmacokinetics, biodistribution and cell uptake of antisense oligonucleotides. Adv Drug Deliv Rev 87: 46–51. 25666165 10.1016/j.addr.2015.01.008

[bib27] Nelson, PS, Kent, M and Muthini, S (1992). Oligonucleotide labeling methods. 3. Direct labeling of oligonucleotides employing a novel, non-nucleosidic, 2-aminobutyl-1,3-propanediol backbone. Nucleic Acids Res 20: 6253–6259. 1475185 10.1093/nar/20.23.6253 PMC334513

[bib28] Soutschek, J, Akinc, A, Bramlage, B, Charisse, K, Constien, R, Donoghue, M et al. (2004). Therapeutic silencing of an endogenous gene by systemic administration of modified siRNAs. Nature 432: 173–178. 15538359 10.1038/nature03121

[bib29] Jeong, JH, Mok, H, Oh, YK and Park, TG (2009). siRNA conjugate delivery systems. Bioconjug Chem 20: 5–14. 19053311 10.1021/bc800278e

[bib30] Harborth, J, Elbashir, SM, Vandenburgh, K, Manninga, H, Scaringe, SA, Weber, K et al. (2003). Sequence, chemical, and structural variation of small interfering RNAs and short hairpin RNAs and the effect on mammalian gene silencing. Antisense Nucleic Acid Drug Dev 13: 83–105. 12804036 10.1089/108729003321629638

[bib31] Yamagami, C, Kawase, K and Iwaki, K (2002). Hydrophobicity parameters determined by reversed-phase liquid chromatography. XV: optimal conditions for prediction of log P(oct) by using RP-HPLC procedures. Chem Pharm Bull (Tokyo) 50: 1578–1583. 12499594 10.1248/cpb.50.1578

[bib32] Valkó, K (2004). Application of high-performance liquid chromatography based measurements of lipophilicity to model biological distribution. J Chromatogr A 1037: 299–310. 15214672 10.1016/j.chroma.2003.10.084

[bib33] Chiu, YL, Ali, A, Chu, CY, Cao, H and Rana, TM (2004). Visualizing a correlation between siRNA localization, cellular uptake, and RNAi in living cells. Chem Biol 11: 1165–1175. 15324818 10.1016/j.chembiol.2004.06.006

[bib34] Berezhna, SY, Supekova, L, Supek, F, Schultz, PG and Deniz, AA (2006). siRNA in human cells selectively localizes to target RNA sites. Proc Natl Acad Sci USA 103: 7682–7687. 16684885 10.1073/pnas.0600148103 PMC1472505

[bib35] Coles, AH, Osborn, MF, Alterman, JF, Turanov, AA, Godinho, BM, Kennington, L et al. (2016). A high-throughput method for direct detection of therapeutic oligonucleotide-induced gene silencing *in vivo*. Nucleic Acid Ther 26: 86–92. 26595721 10.1089/nat.2015.0578 PMC4834514

[bib36] Roehl, I, Schuster, M and Seiffert, S. Oligonucleotide Detection Method. US 20110201006 A1. Application. 2011.

[bib37] Garden, GA and Möller, T (2006). Microglia biology in health and disease. J Neuroimmune Pharmacol 1: 127–137. 18040779 10.1007/s11481-006-9015-5

[bib38] Shitaka, Y, Tran, HT, Bennett, RE, Sanchez, L, Levy, MA, Dikranian, K et al. (2011). Repetitive closed-skull traumatic brain injury in mice causes persistent multifocal axonal injury and microglial reactivity. J Neuropathol Exp Neurol 70: 551–567. 21666502 10.1097/NEN.0b013e31821f891f PMC3118973

[bib39] Brown, GC and Neher, JJ (2010). Inflammatory neurodegeneration and mechanisms of microglial killing of neurons. Mol Neurobiol 41: 242–247. 20195798 10.1007/s12035-010-8105-9

[bib40] Hemmings, HC Jr, Nairn, AC, Aswad, DW and Greengard, P (1984). DARPP-32, a dopamine- and adenosine 3′:5′-monophosphate-regulated phosphoprotein enriched in dopamine-innervated brain regions. II. Purification and characterization of the phosphoprotein from bovine caudate nucleus. J Neurosci 4: 99–110. 6319628 10.1523/JNEUROSCI.04-01-00099.1984 PMC6564759

[bib41] Kordasiewicz, HB, Stanek, LM, Wancewicz, EV, Mazur, C, McAlonis, MM, Pytel, KA et al. (2012). Sustained therapeutic reversal of Huntington's disease by transient repression of huntingtin synthesis. Neuron 74: 1031–1044. 22726834 10.1016/j.neuron.2012.05.009 PMC3383626

[bib42] Kitson, AP, Stark, KD and Duncan, RE (2012). Enzymes in brain phospholipid docosahexaenoic acid accretion: a PL-ethora of potential PL-ayers. Prostaglandins Leukot Essent Fatty Acids 87: 1–10. 22749739 10.1016/j.plefa.2012.06.001

[bib43] Rapoport, SI (2013). Translational studies on regulation of brain docosahexaenoic acid (DHA) metabolism in vivo. Prostaglandins Leukot Essent Fatty Acids 88: 79–85. 22766388 10.1016/j.plefa.2012.05.003 PMC3467358

[bib44] Picq, M, Chen, P, Perez, M, Michaud, M, Véricel, E, Guichardant, M et al. (2010). DHA metabolism: targeting the brain and lipoxygenation. Mol Neurobiol 42: 48–51. 20422316 10.1007/s12035-010-8131-7 PMC2894371

[bib45] Wolfrum, C, Shi, S, Jayaprakash, KN, Jayaraman, M, Wang, G, Pandey, RK et al. (2007). Mechanisms and optimization of *in vivo* delivery of lipophilic siRNAs. Nat Biotechnol 25: 1149–1157. 17873866 10.1038/nbt1339

[bib46] Akinc, A, Zumbuehl, A, Goldberg, M, Leshchiner, ES, Busini, V, Hossain, N et al. (2008). A combinatorial library of lipid-like materials for delivery of RNAi therapeutics. Nat Biotechnol 26: 561–569. 18438401 10.1038/nbt1402 PMC3014085

[bib47] Raouane, M, Desmaële, D, Urbinati, G, Massaad-Massade, L and Couvreur, P (2012). Lipid conjugated oligonucleotides: a useful strategy for delivery. Bioconjug Chem 23: 1091–1104. 22372953 10.1021/bc200422w

[bib48] Kubo, T, Yanagihara, K, Takei, Y, Mihara, K, Morita, Y and Seyama, T (2011). Palmitic acid-conjugated 21-nucleotide siRNA enhances gene-silencing activity. Mol Pharm 8: 2193–2203. 21985606 10.1021/mp200250f

[bib49] Damha, MJ, Giannaris, PA and Zabarylo, SV (1990). An improved procedure for derivatization of controlled-pore glass beads for solid-phase oligonucleotide synthesis. Nucleic Acids Res 18: 3813–3821. 2374710 10.1093/nar/18.13.3813 PMC331081

[bib50] Maier, MA, Yannopoulos, CG, Mohamed, N, Roland, A, Fritz, H, Mohan, V et al. (2003). Synthesis of antisense oligonucleotides conjugated to a multivalent carbohydrate cluster for cellular targeting. Bioconjug Chem 14: 18–29. 12526688 10.1021/bc020028v

