# Supplementary material for: Docosahexaenoic Acid Conjugation Enhances Distribution and Safety of siRNA upon Local Administration in Mouse Brain
Source: Mol Ther Nucleic Acids. 2016 Aug 9;5(8):e344–. doi: 10.1038/mtna.2016.50 (PMC5023396; doi:10.1038/mtna.2016.50)
Supplement: Supplementary Figures and Table [file mtna201650x1.doc]

**Docosahexaenoic acid conjugation enhances distribution and safety of siRNA upon local administration in mouse brain**

**Short title: DHA-hsiRNAs silence *Htt* in neurons and mouse brain**

Mehran Nikan^1,2,#^, Maire F. Osborn^1,2,#^, Andrew H. Coles^1,2,#^, Bruno M. D. C. Godinho^1,2^, Lauren M. Hall^3^, Reka A. Haraszti^1,2^, Matthew R. Hassler^1,2^, Dimas Echeverria^1,2^, Neil Aronin^1,3^, Anastasia Khvorova^1,2^

*^1^ RNA Therapeutics Institute, University of Massachusetts Medical School*

*^2^ Department of Molecular Medicine, University of Massachusetts Medical School*

*^3^ Department of Medicine, University of Massachusetts Medical School*

Correspondence should be addressed to A.K. ([Anastasia.khvorova@umassmed.edu](mailto:Anastasia.khvorova@umassmed.edu))

This work was done in Worcester, Massachusetts, United States of America.

# These authors contributed equally to this work.

Corresponding Author:

Anastasia Khvorova (Anastasia.khvorova@umassmed.edu; 774-455-3638 / 508-856-6696 (fax)), University of Massachusetts Medical School, 368 Plantation Street, Worcester, MA, 01605


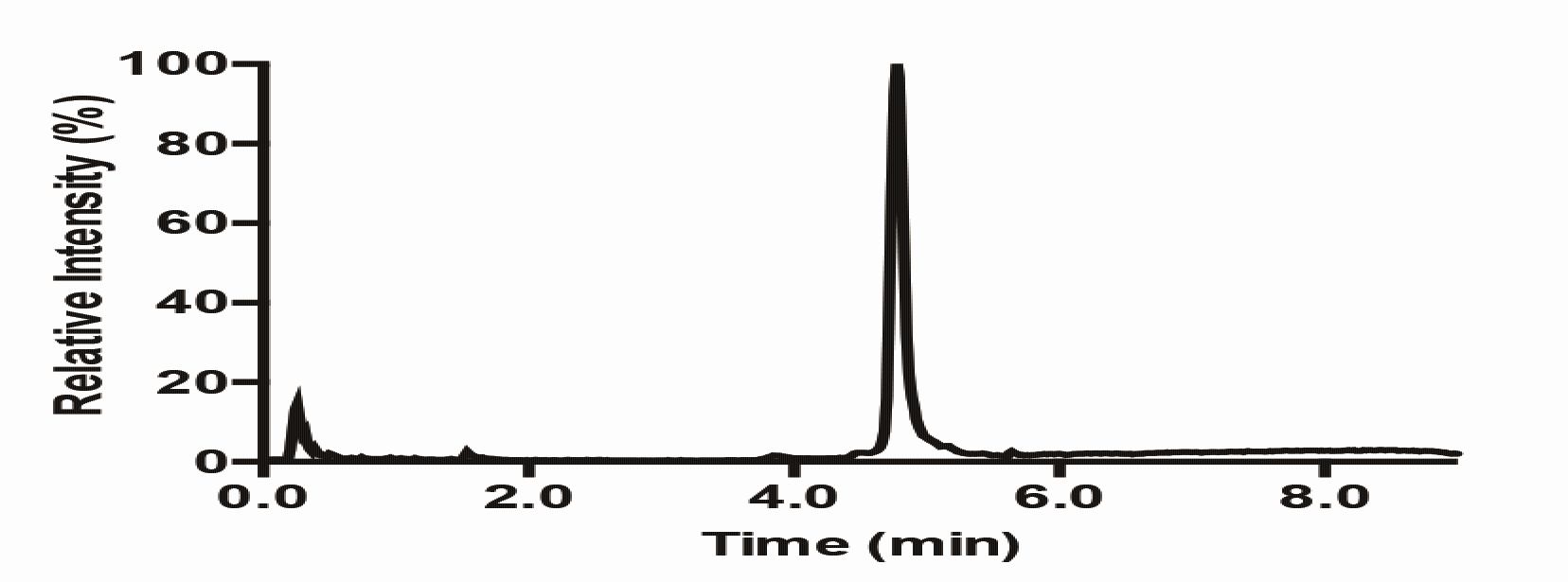
**Supplementary Fig. 1. Representative LC-MS characterization of Cy3-DHA-hsiRNA*^HTT^.*** Calculated: 6174.1 for [M-H]^-^, found: 6174.4. Conditions: Buffer A: 15mM Dibutylamine/25mM HFIP, Buffer B: 20%A in MeOH, Column: xbidge OST C18, 2.5 um, Gradient: 0-10 min (1% B- 80% B), 10-13 min (80% B- 80% B), 13.0-13.1 min (80% B- 1% B), 13.1-18 min (1% B -1% B).

**Supplementary Table 1. Modified Oligonucleotide Sequences.** Chemical modifications are abbreviated as follows, where “X” corresponds to A, U, G, or C: fX (2ʹ-fluoro), mX (2ʹ-O-methyl), P (5ʹ-phosphate), Chol (Cholesterol), ‘#’ (phosphorothioate backbone modification), ‘.’ (phosphodiester backbone). Oligonucleotide synthesis procedures are described in Materials and Methods.

**Supplementary Fig. 2. DHA-hsiRNA*^PPIB^* silences Cyclophilin B (*Ppib*) mRNA in mouse primary cortical neurons.** Primary cortical neurons were incubated with Chol-hsiRNA*^PPIB^*, DHA-hsiRNA*^PPIB^*, and DHA-hsiRNA^NTC^ at concentrations shown for one week. Level of huntingtin mRNA was measured using QuantiGene® (Affymetrix) normalized to housekeeping gene, *Hprt* (hypoxanthine phosphoribosyltransferase), and presented as percent of untreated control (n=3, mean +/- SD). UNT – untreated cells.

**Supplementary Fig. 3. Non-conjugated hsiRNA*^HTT^* exhibits modest Huntingtin (*Htt*) mRNA silencing in mouse primary cortical neurons.** Primary cortical neurons were incubated with hsiRNA*^HTT^* at concentrations shown for one week. Level of huntingtin mRNA was measured using QuantiGene® (Affymetrix) normalized to housekeeping gene, *Hprt* (hypoxanthine phosphoribosyltransferase), and presented as percent of untreated control (n=3, mean +/- SD). UNT – untreated cells. Results shown from two separate experiments.

**Supplementary Fig. 4. DHA-hsiRNA*^PPIB^* silences Cyclophilin B (*Ppib*) mRNA in mouse striatum following a single intrastriatal injection.** DHA-hsiRNA*^PPIB^* (60 µg) and DHA-hsiRNA^NTC^ (60 µg) were bilaterally injected into the striatum of WT mice. The left half of the brain was collected after 7 days and punch biopsies of the straitum were processed for mRNA level quantification (see **Materials and Methods**). Level of *Ppib* mRNA was measured using QuantiGene® (Affymetrix) normalized to housekeeping gene, *Hprt* (hypoxanthine phosphoribosyltransferase), and presented as percent of untreated control (n=5 mice, mean ± SD). NTC = non-targeting control; CSF = artificial cerebrospinal fluid (**** = p<0.0001).

**Supplementary Fig. 5. DHA-hsiRNA*^PPIB^* silences Cyclophilin B (PPIB) protein in mouse striatum following a single intrastriatal injection.** DHA-hsiRNA*^PPIB^* (60 µg) and DHA-hsiRNA^NTC^ (60 µg) were bilaterally injected into the striatum of WT mice. The right half of the brain was collected after 7 days and punch biopsies of the straitum were processed for protein level quantification (see **Supplementary Materials and Methods**). Level of PPIB protein expression was analyzed by western blot and compared to a loading control (β-actin). NTC = non-targeting control; CSF = artificial cerebrospinal fluid.

**Supplementary Materials and Methods**

**Protein level quantification from tissue punches**

Protein levels were quantified from tissue punches (3 mm) collected and snap-frozen 7 days after a bilateral intrastratal injection (see **Materials and Methods**). Tissue biopsies were thawed into 1X RIPA buffer (Sigma Aldrich) with cOmplete™ protease inhibitor cocktail (Roche) and homogenized using a TissueLyser II (Qiagen) at 30 hz for 8 minutes, followed by a one minute incubation in a water bath sonicator.  Samples were incubated on ice for 15 minutes and subsequently centrifuged at 13,000 rpm at 4°C for 10 minutes.  50 µg of protein per sample was loaded in a Bolt 4-12% Bis-Tris gradient gel (Life Technologies) and run at 165 volts for 35 minutes. Protein bands were then transferred onto a PVDF membrane (Bio-Rad Trans-Blot Turbo).  The membrane was incubated in primary and secondary antibodies overnight in an iBind cassette (Life Technologies).  The antibodies used were beta-actin anti-Rabbit (Abcam #ab8227; 1:667), Cyclophilin B (PPIB) anti-Mouse (Abcam #74173; 1:200), anti-mouse (Bio-rad; 1:667), and anti-rabbit (Bio-rad; 1:667).  After 24 hours, the membrane was developed using a Bio-rad imaging system and Clarity™ Western ECL reagent.
